# The Yeast Spore Wall Enables Spores to Survive Passage through the Digestive Tract of *Drosophila*

**DOI:** 10.1371/journal.pone.0002873

**Published:** 2008-08-06

**Authors:** Alison E. Coluccio, Rachael K. Rodriguez, Maurice J. Kernan, Aaron M. Neiman

**Affiliations:** 1 Department of Biochemistry and Cell Biology, Stony Brook University, Stony Brook, New York, United States of America; 2 Department of Neurobiology and Behavior, Center for Developmental Genetics, Stony Brook University, Stony Brook, New York, United States of America; Texas A&M University, United States of America

## Abstract

In nature, yeasts are subject to predation by flies of the genus *Drosophila*. In response to nutritional starvation *Saccharomyces cerevisiae* differentiates into a dormant cell type, termed a spore, which is resistant to many types of environmental stress. The stress resistance of the spore is due primarily to a spore wall that is more elaborate than the vegetative cell wall. We report here that *S. cerevisiae* spores survive passage through the gut of *Drosophila melanogaster.* Constituents of the spore wall that distinguish it from the vegetative cell wall are necessary for this resistance. Ascospores of the distantly related yeast *Schizosaccharomyces pombe* also display resistance to digestion by *D. melanogaster*. These results suggest that the primary function of the yeast ascospore is as a cell type specialized for dispersion by insect vectors.

## Introduction

In the absence of nitrogen and the presence of a non-fermentable carbon source, diploid cells of the yeast *Saccharomyces cerevisiae* undergo meiosis and the resulting haploid nuclei are packaged into spores [1]. Spores are quiescent cells that display resistance to a variety of environmental insults. *S. cerevisiae* spores are characterized by a thick coat, or spore wall, that is more extensive than the cell wall of vegetative cells and this spore wall is essential for the resistance of the spores to environmental stress [2]. The spore wall is composed of four layers of different polymers [2]. The two inner layers consist primarily of mannoproteins and beta-glucans, and are similar to the walls of vegetative cells [3]. The third and fourth (outermost) layers are specific to the spore and are composed, respectively, of chitosan and of a dityrosine-containing polymer [4], [5]. The enhanced resistance of the spore to many stresses is attributable to these two outer wall layers [6], [7]. In hemiascomycete yeasts such as *Saccharomyces* spores commonly form in a set of four, termed a tetrad, that are enclosed within a sac, termed an ascus [8].

Filamentous fungi often form elaborate structures to assist in the wind-driven dispersal of (asexual) conidiospores or ascospores [9], [10]. Yeast produce no such structures and it has been suggested that the ascospores are primarily a survival form rather than a dispersal form [9]. Spores have been shown to be resistant to laboratory treatments such as exposure to ether vapor or temperature shock at 55°C [6], [11], but the relevance of these treatments to stresses in the natural environment is unclear. The use of yeasts as a food source by Drosophilid species in the wild is well documented [12]. Previous laboratory studies with *S. cerevisiae and D. melanogaster* indicate that vegetative cells are killed by passage through the gut and that spores have increased survival, but this has not been rigorously quantitated [13], [14]. We report here direct evidence that spores display enhanced survival relative to vegetative cells in passage through the gut of *Drosophila melanogaster*, and that mutations specifically affecting the spore wall reduce their survival rate. Moreover, resistance requires the layers unique to the spore wall. These data suggest that *S. cerevisiae* ascospores are a cell type specialized for dispersal in the environment via *Drosophila* vectors.

## Results

### Spores are resistant to stresses associated with predation

**Figure 1:**
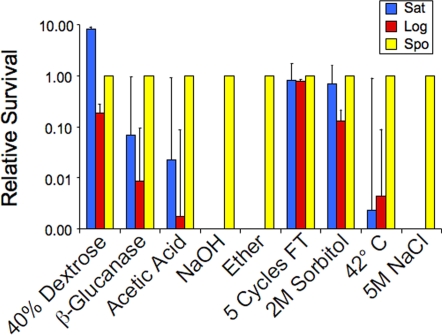
Relative survival of stationary phase cells, log phase cells and spores to different stresses. The survival of vegetative cells from a saturated culture (Sat), a log phase culture (Log), or spores (Spo) after exposure to various stresses was measured as described in the Methods. For each condition, at least three independent experiments were performed and the average percent survival determined. For the graph, the survival rate of spores was defined as 1 and the relative survival of the vegetative cultures is shown. Thin lines represent the range of relative survival. The average percent survivals of spores under each condition were: 40% dextrose, 60%; β-glucanase, 244%; Acetic acid, 54%; NaOH, 23%; Ether 52%; Freeze/Thaw 94%; 2M Sorbitol, 76%; 42°C, 73%; 5M NaCl, 51%.

Because one function of the spore is thought to be to allow persistence in the environment, we examined the survival of spores in a variety of treatments mimicking natural stresses. In this analysis, spores were compared to vegetative cells in two phases of growth: log phase cultures containing actively budding cells and stationary phase cultures (Figure 1). Stationary phase cells provide a particularly good comparison because, like spores, they are unbudded, quiescent cells but lack the spore wall outer layers. Relative to log phase cells, spores were more resistant to all the stress treatments. However, stationary phase cells were as resistant as spores to some of the stressors, in particular, those stresses meant to mimic weather conditions. Stationary phase cells were as competent as spores at surviving repeated freeze thaw cycles and increased osmolarity, either with high levels of dextrose or with sorbitol. Additionally, stationary phase cells were comparable to spores in qualitative assays for survival of desiccation (data not shown).

However, spore walls are essential for specific types of stress resistance. As previously reported, spores were more resistant than vegetative cells to ether vapor and to treatment with glucanases [11], [15]. Spores are also known to be resistant to short periods of heat shock at 55°C [6], and we found that they similarly survive extended incubations at 42°C. In addition, we found that spores were more resistant than stationary phase cells to very high salt concentrations and exposure to high or low pH. Because spore walls are essential for stress resistance, and contain chitosan and dityrosine layers not found in the vegetative wall, resistance to high salt and pH extremes is likely a property of the chitosan and/or dityrosine layers, as has been shown for ether and zymolyase resistance [6], [7]. While the ecological significance of resistance to ether vapor or 5M salt is not immediately obvious, in the environment yeast cells likely are exposed to acidic or basic conditions as well as degradative enzymes either as a consequence of exposure to other microorganisms or ingestion by animals [16]–[18]. Thus, these results suggest that the specialized function of the spore wall is not resistance to environmental stresses *per se*, but rather survival in the face of competition or predation by other organisms.

### Spores survive passage through the *Drosophila* gut

**Figure 2:**
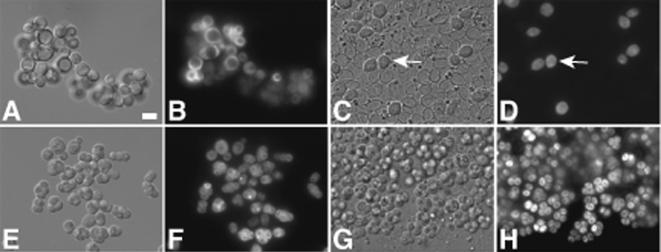
Spores are intact in *Drosophila* frass. Vegetative cells or spores of strain AN390 were fed to *Drosophila* and the frass was analyzed by DIC and fluorescence microscopy. A) DIC image of vegetative cells before ingestion. B) Fluorescence image of cells in A. C) DIC image of a flyspeck from *Drosophila* fed vegetative cells. Arrow indicates an intact vegetative cell. D) Fluorescence image of cells in C. E) DIC image of spores before ingestion. F) Fluorescence image of spores in E. G) DIC image of a flyspeck from *Drosophila* fed spores. H) Fluorescence image of cells in G. Scale bar = 5 microns.

To test this possibility, we established an assay to quantify the survival of *S. cerevisiae* after ingestion and passage through the gut of the fruit fly *D. melanogaster*. The insect midgut is reported to have regions of both high and low pH [19], conditions that might select for spores over stationary phase cells (Figure 1). For our assay, we constructed strains in which the *TEF2* gene, encoding translation elongation factor 2a, an abundant cytoplasmic protein, was tagged with GFP. Intact cells of this strain display bright cytoplasmic fluorescence (Figure 2B). *Drosophila* were starved for six hours and then placed into a petri dish with either stationary phase cells or spores carrying the *TEF2::GFP* reporter. A cover slip was attached to the petri dish lid. After 18 hours, the cover slip was placed on a slide and individual excreta (flyspecks) were visualized directly in the fluorescence microscope. Intact cells retained their cytoplasmic fluorescence, while dead cells were no longer fluorescent. This assay allows for quantitation of cell survival, and because the cells were directly visualized in the feces (frass), ensures that they have passed through the gut rather than having been transferred to the cover slip from the exterior of the fly.

By differential interference (DIC) microscopy, most of the stationary phase cells in the frass appear to be ghosts with intact walls empty of contents (Figure 2C). Consistent with this, the ghost cells lack cytoplasmic fluorescence (Figure 2D). By contrast, the majority of spores in the frass appear intact both by DIC and fluorescence, indicating that they are resistant to digestion in the fly gut (Figure 2G, H). The spores are still clustered in sets of three or four, suggesting that spores from individual asci tend to hold together during passage. However, the ascal sac is missing in most cases, indicating that the spores are held together by the interspore bridges which connect the spore walls [20].

For each condition, images were collected from multiple flyspecks and the percent survival calculated as the fraction of intact cells, as judged by the presence of a fluorescence signal (Table 1). For stationary phase cells, the percent survival in different flyspecks ranged from <1% to 20% with an average survival of 8%. As any cells that were killed and digested beyond recognition in the microscope would not be counted, these numbers represent the upper limit of survival. The percent survival of spores also varied between flyspecks with a low of 20% to greater than 99% of the spores retaining cytoplasmic fluorescence. On average, 87 % of the spores survived intact in the frass. These results demonstrate that spores survive passage through *Drosophila* significantly more efficiently than vegetative cells.

**Table 1:** Quantitation of cell survival in frass.

Cell type	Relevant Genotype^1^	Average survival^2^ (%+/−SD)
Vegetative	WT	8+/−7
Spores	WT	87+/−14
Spores	*dit1*	30+/−21
Spores	*mum3*	8+/−2
Spores	*osw1*	3+/−3
*S. pombe* vegetative	WT	3+/−4
*S. pombe* spores	WT	38+/−14
Spores	*yfr039c*	37+/−16
Spores	*yjl037w*	21+/−11

1 Strains used; *S. cerevisiae* WT is AN390, *dit1* is AN391, *S. pombe* is YDM124.

2 For each condition, at least 9 flyspecks were photographed and survival in each was quantified by counting >100 cells.

### The unique layers of the spore wall are necessary for spore survival

**Figure 3:**
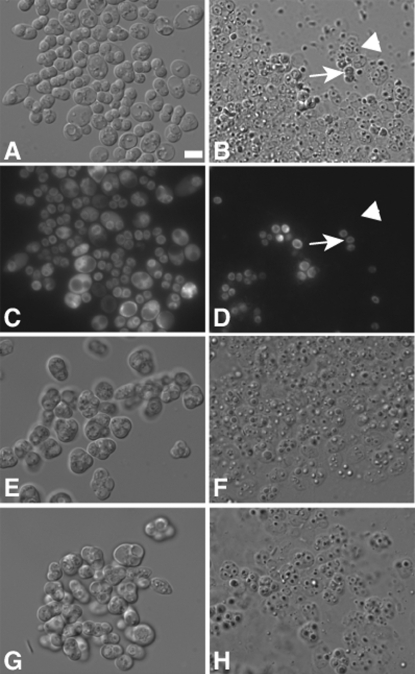
Spores with defective spore walls display reduced survival in frass. Spores mutant for *dit1*, *mum3*, or *osw1* were fed to *Drosophila* and the frass was analyzed by DIC and fluorescence microscopy. A) DIC image of *dit1* spores before ingestion. B) DIC image of *dit1* spores in frass. Arrow indicates an intact spore. Arrowhead indicates a lysed spore. C) Fluorescence image of spores in A. D) Fluorescence image of spores in B. E) DIC image of *mum3* spores before ingestion. F) DIC image of *mum3* spores after ingestion. G) DIC image of *osw1* spores before ingestion. H) DIC image of *osw1* spores in frass.

To examine if the spore wall is important for resistance to digestion, strains lacking *DIT1, OSW1, or MUM3* were examined. *DIT1* encodes an enzyme required for synthesis of the outermost dityrosine layer of the spore wall while in the absence of *OSW1* or *MUM3* both the chitosan and dityrosine layers are lost [21]. Frass from flies fed spores of the *dit1* strain displayed an increased proportion of apparent spore ghosts, which again correlated well with the loss of cytoplasmic fluorescence in the spores (Figure 3B, D). Quantitation revealed that *dit1* spores were more sensitive than wild-type spores, but still more resistant than stationary phase cells, with an average survival of 30%. The *osw1* and *mum3* spores appeared even more sensitive than *dit1*, with the spore ghosts difficult to distinguish and cellular debris apparent in the frass (Figure 3F, H). Survival in these strains, quantitated on the basis of DIC appearance rather than fluorescence, was only 3% and 8%, respectively, comparable to the survival of stationary phase wild type cells (Table 1). Taken together, these data indicate that the dityrosine layer is important, and the chitosan and dityrosine layers together are essential, for the resistance of spores to digestion by *Drosophila.*

If a significant fraction of sensitive cells were digested beyond recognition in the microscope, they would be missed in this fluorescence assay and the calculated survival would represent an overestimate of the true rate of cell survival. As an alternative assay, we compared the survival of wild type spores to that of *dit1* spores, *osw1* spores, or stationary phase cells by feeding mixed cultures to *Drosophila*, similar to what has been described previously [13]. The ratio of the two cell types in the mixes was determined by titering the mixture on plates selective for either the wild type or other cells both before feeding and after resuspension of the frass (see Materials and Methods). Their enrichment or depletion in the frass provides a measure of the survival efficiency of the mutants relative to wild type spores (Table 2). By this assay, wild type spores survive passage through the gut ∼6-fold more often than *dit1* spores, 14-fold more often than *osw1* spores and 42-fold more often than stationary phase vegetative cells. These numbers are in good agreement with ratios derived from the direct measurements of survival in the fluorescence assay (Table 1), with the exception that the apparent survival of vegetative cells is somewhat lower in this assay. These results confirm the importance of the outer spore wall layers in resistance to digestion and, interestingly, suggest that walls of most spores and vegetative cells that are killed during passage through the gut remain sufficiently intact to be visible in the light microscope.

**Table 2:** Competitive survival assays^1^

Cell type tested	Relevant Genotype^2^	Survival Ratio (WT : tested strain)^3^
Spores	*dit1*	5.6
Spores	*osw1*	14.3
Vegetative	WT	42.5

1 For each cell type tested, cultures were mixed with WT spores and the survival in the frass relative to the WT was calculated as described in Methods.

2 Strains used; *S. cerevisiae* WT spores, NKY895; *dit1*, AN264; WT vegetative cells, AN117-4B.

3 Ratios are the average of four experiments.

### *S. pombe* spores resist digestion by *Drosophila*

**Figure 4:**
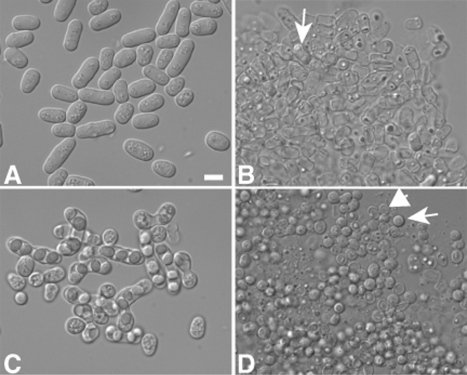
*S. pombe* spores display increased resistance to passage through *Drosophila*. Vegetative or sporulated cells of strain YDM124 were fed to *Drosophila* and flyspecks were analyzed by light microscopy. A) Vegetative cells before ingestion. B) Vegetative cells in frass. Arrow indicates an intact cell. C) Spores before ingestion. D) Spores in frass. Arrow indicates an intact spore. Arrowhead indicates a lysed spore.

To determine if resistance to digestion was unique to *S. cerevisiae* spores, we examined the survival of vegetative cells and ascospores of the distantly related yeast *Schizosaccharomyces pombe* . The spore wall of *S pombe* is also more elaborate than its vegetative cell wall and, like the *S cerevisiae* spore wall, confers resistance to organic compounds [22]–[24]. However, *S. pombe* spore walls are different in composition from *S. cerevisiae*; for instance, though they may contain chitosan they lack dityrosine [22], [25], [26]. As in *S. cerevisiae*, vegetative cells of *S. pombe* were sensitive to digestion by *Drosophila* and spores displayed increased survival (Figure 4), though in both forms, *S. pombe* was somewhat more sensitive than *S. cerevisiae* to digestion (Table 1). These results suggest that resistance to digestion is a common feature of yeast ascospores and raise the possibility that *S. cerevisiae* may be somewhat better adapted for dispersal by *D. melanogaster* than is *S. pombe*.

### *S. cerevisiae* genes required for survival in the gut

If the spore wall has been adapted specifically for resistance to digestion, we might expect to find genes that are required for digestion resistance but not necessary for resistance to other stresses. To examine this possibility, we screened yeast strains from a collection deleted for genes transcriptionally induced during sporulation [27]. This collection has been previously analyzed for mutants effecting meiotic chromosome segregation, spore formation, and ether resistance [21], [27]. We analyzed ∼250 individual strains. Each strain was sporulated and the spores were fed to *Drosophila*. Because of the variability in survival of wild-type spores in different flyspecks, multiple flyspecks were examined by DIC microscopy for each mutant and survival was quantified by counting the ratio of intact to ghost spores. All strains that had previously been reported to be defective in spore formation [27] were very sensitive to digestion. Similarly, mutants with previously reported defects in the spore wall [21], such as *osw1* and *mum3*, were sensitive to passage through the gut.

We were particularly interested in strains in which no evident defects were noted in previous screens. About 20 such mutant strains displayed low survival (<20%) in our initial screen and were retested. Ultimately, two ORFs, *YJR037w* and *YFR039c*, were identified in which mutations caused reproducibly lower average survival (21% and 37%, respectively) than wild type. While modest, this sensitivity is comparable to that of mutants lacking the dityrosine layer (Table 1). But, unlike *dit1* cells, these mutants are resistant to ether vapor [21]. The predicted sequences of the two proteins do not contain any conserved motifs that might indicate their function. However, both proteins are predicted to be secreted, suggesting that they could be components of the spore wall important for survival in the gut.

## Discussion

The results presented here demonstrate that although entry into stationary phase is sufficient to allow vegetative yeast cells to resist many stresses, the spore wall confers additional resistance, particularly to stresses associated with ingestion. The ability to survive passage through the *Drosophila* gut is greatly enhanced by the unique chitosan and dityrosine layers of the spore wall.

Spores form in response to starvation. By enabling the spores to “travel” in the gut of the fly, the spore wall allows for the saltatory dispersal of cells to distant niches. This function of the spore wall is analogous to the way the coats of many seeds allow them to be dispersed by avian or animal vectors [28]. A field study of *D. melanogaster* infestation of figs found that, while laying eggs, the flies introduce the yeasts that will eventually rot the fruit [18]. While adhesion of the yeast to exterior of the fly is one possible means of such transport, our results suggest that the spores may be delivered in frass deposited at the same time as the eggs. This dispersion mechanism may be more effective than wind or water-mediated forms in that it recruits the chemosensory and locomotor abilities of the fly, so that dispersal is directed to nutrient-rich environments.

If ascospores are primarily adapted to function in dispersal, why couple their formation to meiosis? Indeed, in filamentous fungi, formation of asexual conidiospores is a common dispersal strategy. The ability of spores to survive passage through the *Drosophila* gut has been shown to promote outbreeding, that is, mating between spores from different asci [13], [29]. It has been proposed that coupling the acquisition of mating competence (return to haploidy) to dispersal may be a strategy for maintaining genetic diversity in the population [13]. Our finding that the unique structures of the spore wall provide the resistance necessary for passage through the gut is consistent with this hypothesis. Moreover, meiotic recombination prior to spore formation ensures that, even without outbreeding, genetic diversity in the spore population is higher than in the precursor vegetative population. Increasing genetic diversity of the population prior to dispersal increases the chances for selection of more optimal genotypes in the new environments to which the yeast are dispersed.

A number of studies have described associations between specific insects and fungi, including between particular species of *Drosophila* and of budding yeasts [12], [30]–[32]. It is possible that as part of these associations the yeast partner in such a pair will have become adapted to its specific insect vector. For instance, the greater resistance of *S. cerevisiae* than *S. pombe* to digestion in our tests may indicate that the natural insect vector for *S. cerevisiae* is more closely related to *D. melanogaster* than the *S. pombe* vector. The spore coats of hemiascomycetous yeast are frequently elaborately shaped and these forms have been used for taxonomic classification [33]. The reason for these elaborations is not known, though in light of our results, they may represent adaptations that allow for more efficient dispersal by specific insect species. It will be of interest to determine if, perhaps, particular yeast species are better adapted for survival in the particular *Drosophila* species with which they are associated with in the wild.

Although the spores can pass through the gut intact, the ascus sac appears to be removed in the process. The disappearance of the sac allows contact of spores from different asci and would aid outbreeding [13], but also raises some intriguing questions. The wall of the ascus is derived from the cell wall and is thought to be of similar composition, yet our data indicate that the vegetative wall, though not the cell inside, is intact after passage. These results reveal an unknown difference between the cell and ascal walls. A large fraction of the cytoplasm and organelles of the original cell remain behind in the ascus [34]. It is possible that, as with berries distributed by birds, the ascal wall and contents provide some nutritional value for the fly so that the consumption and dispersal of spores by flies is beneficial to both organisms. The interactions of flies and yeasts might therefore be mutualistic as for frugivores and fruiting plants [28], [35].

## Materials and Methods

### Strains and Media

Standard yeast media and genetic methods were used [36]. The wild type strain used for the stress tests was K8409 [27]. *Drosophila* were reared on standard agar/molasses/yeast medium [37], but were starved, with only water available, before feeding experiments. For the *Drosophila* feeding experiments, three different wild-type *Drosophila* stocks were used; Canton S, Oregon-R and Oregon-RS, all obtained from the Bloomington *Drosophila* stock center, Bloomington, IN. Similar results were obtained with all three strains. The yeast strains AN390 (*MAT* ***a*** */MATα ura3/ura3 trp1/trp1 his3/his3 TEF2::GFP ::his5^+^/TEF2::GFP::his5* ^+^) and AN391 (*MAT* ***a*** */MATα ura3/ura3 his3/his3 TEF2::GFP ::his5^+^/TEF2::GFP::his5* ^+^ *dit1 ::his5^+^/dit1::his5^+^*) were constructed by outcrossing a *TEF2::GFP* tagged *MAT* ***a*** strain [38]to haploids AN117-4B [39]and AN263-5A (as AN117-4B, plus *dit1::his5^+^*) in the fast-sporulating SK-1 background [40], and crossing the resulting segregants. The *mum3* and *osw1* strains have been described [27]. The wild type (NKY895) and *dit1* (AN264) strains used for the competitive survival assays have also been described elsewhere [20], [41]. The *S. pombe* strain, YDM124 (*h90*) was provided by Dan McCollum (U Mass Worcester).

### Stress Treatments

To analyze resistance to different stresses, log phase, stationary phase or spores of strain were prepared. For log phase cells, an overnight culture in YPD was diluted 1∶25 into fresh YPD medium and grown for 3 hours. Stationary phase cells were from a culture grown to saturation in YPD. Spores were prepared by incubation in liquid sporulation medium until the culture contained greater than 70% asci. For ether treatment, cells in culture medium were diluted 1∶2 with ethyl ether, mixed, and after 10 minutes, samples were removed, diluted, and titered. For treatment with 1% sodium hydroxide, 50 microliters of the cells in culture medium were diluted into 450 microliters of 1% sodium hydroxide, incubated for 10 minutes and then titered. Acetic Acid treatment was performed as for sodium hydroxide, except that cells were placed in a 2% acetic acid solution for 20 minutes. To test Zymolyase sensitivity, Zymolyase 100 T (US Biologicals) was added to the cells in culture medium to a final concentration of 0.4 mg/ml and then incubated at 37°C for 1 hr before titering. To test osmolarity, cells were diluted 1∶10 into 2M Sorbitol, incubated overnight at room temperature and then titered. Sensitivity to high salt concentration was tested similarly except that cells were diluted into 5M NaCl. To examine resistance to freeze thaw cycles, 1 ml of cell culture was frozen by incubation at −20° and the thawed by incubation at room temperature. This was repeated five times before cells were plated for titer. In all cases, survival was calculated as the titer of cells after treatment divided by the viable cell titer of the culture before treatment. To assay dessication, cells from log phase, stationary phase, or sporulated cultures were dried onto a paper filter and incubated at room temperature for five days. The cells were then rehydrated by placing the filter onto a YPD plate, the cells were replica plated onto a second YPD plate and survival assessed by growth on the replica plate.

### Fluorescence assays of passage through *Drosophila*

For *S. cerevisiae*, patches of strains to be tested were incubated on YPD plates or SPO plates and then a sterile toothpick was used to make a patch of the yeast on the agar surface of a 50 mm petri dish containing 10 ml of 2% agar. Two 22 mm cover slips were adhered to the underside of the lid of the petri dish using 1 microliter of sterile water. Flies to be used in the experiment were first starved in a humidity chamber for >6 hrs to allow them to empty gut contents. Twelve to fifteen flies were then placed in each petri dish with the yeast, and the plates were left at room temperature. After overnight incubation, the flies were removed and individual excreta on the cover slips examined by light and fluorescence microscopy with a Zeiss Axioplan 2 microscope. Images were collected using a Zeiss mRM Axiocam and AxioVision 5.1 software. For each flyspeck >100 cells were scored as intact or not and survival was calculated as the percentage of cells that appeared intact. To calculate an average survival, at least 9 different flyspecks were examined. For strains AN390 and AN391, scoring of cells as intact or not intact was determined by the presence or absence of cytoplasmic *TEF2::GFP* fluorescence. As this correlated very strongly with the cells' appearance in DIC, survival of the other strains assayed was scored directly in DIC.

### Comparative survival assays

A sporulated wild type culture (NKY895) was mixed with tester cultures: sporulated *dit1* cells (AN264), *osw1* spores, or stationary phase cells (AN117-4B). All sporulated cultures were >80% asci. To determine the input ration of wild type : tester cells, the mixes were titered on both TRP (selective for wild type) and ADE (selective for tester strain) dropout media. The mixed cultures were pelleted, spotted onto agar in a 50 mm petri dish and flies were introduced as described above. To reduce contamination, flies were raised on sterile apple juice medium for >2 days prior to the experiment. After overnight incubation, a cover slip was removed from the petri dish lid, cut in half, and vortexed in 1 ml of water in a 15 ml conical tube. Serial dilutions were again plated on ADE and TRP dropout media to determine the titer of the wild type and tester strains. Division of the endpoint wild type : tester ratio by that in the starting culture produces the calculation of the relative survival efficiency of wild type spores shown in Table 2.
